# Cytotoxic Effects of *Artemisia annua* L. and Pure Artemisinin on the D-17 Canine Osteosarcoma Cell Line

**DOI:** 10.1155/2019/1615758

**Published:** 2019-07-04

**Authors:** Gloria Isani, Martina Bertocchi, Giulia Andreani, Giovanna Farruggia, Concettina Cappadone, Roberta Salaroli, Monica Forni, Chiara Bernardini

**Affiliations:** ^1^Department of Veterinary Medical Sciences, University of Bologna, Ozzano Emilia, 40064 Bologna, Italy; ^2^Department of Pharmacy and Biotechnology, University of Bologna, 40127 Bologna, Italy

## Abstract

*Artemisia annua* has been used for centuries in Traditional Chinese Medicine. Although used as an antimalarial drug, its active compound artemisinin and the semisynthetic derivatives have also been investigated for their anticancer properties, with interesting and promising results. The aims of this research were to evaluate (i) the cytotoxicity and the antiproliferative effect of pure artemisinin and a hydroalcoholic extract obtained from *A. annua* on the D-17 canine osteosarcoma cell line and (ii) the intracellular iron concentration and its correlation with the cytotoxic effects. Both artemisinin and hydroalcoholic extract induced a cytotoxic effect in a dose-dependent manner. Pure artemisinin caused an increase of cells in the S phase, whereas the hydroalcoholic extract induced an evident increase in the G_2_/M phase. A significant decrease of iron concentration was measured in D-17 cells treated with pure artemisinin and hydroalcoholic extract compared to untreated cells. In conclusion, although preliminary, the data obtained in this study are indicative of a more potent cytotoxic activity of the hydroalcoholic extract than pure artemisinin, indicating a possible synergistic effect of the phytocomplex and a mechanism of action involving iron and possibly ferroptosis. Considering the similarities between human and canine osteosarcomas, progress in deepening knowledge and improving therapeutic protocols will probably be relevant for both species, in a model of reciprocal translational medicine.

## 1. Introduction

Since ancient time, *Artemisia annua* L. has been used as a medicinal plant for the treatment of several diseases in Traditional Chinese Medicine [1]. To date, the reputation of *A. annua* is linked to its antimalarial activity. In 2015, Youyou Tu was awarded the Nobel Prize for isolating the active molecule artemisinin from *A. annua*. Currently, artemisinin, its derivatives, and Artemisinin Combination Therapy (ACT) belong to the established standard treatments of malaria worldwide [2]. Over 600 phytochemicals have been identified as constituents of *A. annua*, but its phytochemistry is dominated by sesquiterpenoids, flavonoids, and coumarins, together with enzymes (such as *β*-galactosidase and *β*-glucosidase) and steroids (e.g., *β*-sitosterol and stigmasterol) [1]. However, *A. annua* is distinguished from the other 200 species of the *Artemisia* genus by the exclusive presence of artemisinin, the active compound present in dried leaves (WHO monograph) [3]. Artemisinin is a sesquiterpene trioxane lactone, which contains an endoperoxide bridge essential for its biological activity. The low amount of artemisinin extracted from the plant, its low hydro- and liposolubility, and its limited bioavailability can represent a serious limitation for the standardization and commercialization of the drug. In the last 30 years, several semisynthetic artemisinin derivatives were developed with different strategies, including genetic engineering [1, 4, 5]. Although developed as antimalarials, artemisinin and its semisynthetic derivatives have also been investigated for many other therapeutic properties, such as antiviral, antimicrobial, and anti-inflammatory activities [6]. However, since the late 1990s, anticancer properties of artemisinins have been well known and there has been a rapid increase of *in vitro* and *in vivo* studies, case reports, and clinical trials on the antitumor properties of artemisinins [7]. The endoperoxide moiety is strategic for the bioactivity of artemisinin-type drugs. Its cleavage leads to reactive oxygen species (ROS) formation and induces oxidative stress. Furthermore, in the presence of ferrous iron or reduced heme, artemisinin can convert itself into cytotoxic carbon-centered radical, a highly potent alkylating agent, to induce direct oxidative damage to cancer cells [2, 7]. Indeed, it has been reported that artemisinins induce apoptosis and ferroptosis, reduce cell proliferation through cell cycle arrest, and inhibit angiogenesis and tissue invasion of the tumor, as well as cancer metastasis [2, 6, 7].

Spontaneously occurring osteosarcoma (OSA) in dogs has clinical presentation, biological behaviour, response to treatment, and disease progression similar to human OSA [8–10]. OSA is the most common primary malignancy of bone both in dogs and humans. It is significantly more prevalent in dogs, with an incidence rate 27 times higher in dogs than in people. OSA commonly occurs in old dogs (median age 7 years), while in humans is more common in adolescence (10- to 14-year-old age group) [8, 11]. The site of OSA development in children and dogs is strikingly similar, with a predilection for the weight-bearing region of long bones. Approximately 75% of canine OSA occurs in the appendicular skeleton, with the most common sites in the distal radius and proximal humerus. In human OSA, long bones are affected in up to 90% of cases, with the distal femur, proximal tibia, and the proximal humerus being the most common locations [9, 11]. Furthermore, mutations of specific genes involved in the etiopathogenesis of OSA are found in both species, including mutations in the tumor suppressor genes p53, RB1, and PTEN and alterations of the oncogenes MYC and MET [8]. However, therapeutic failures are recurrent in both dogs and humans and are mainly due to the development of multiple resistance and metastatic spread, making the development of new therapies essential.

Therefore, to provide new scientific evidence to support anticancer activity of *A. annua* L., the first purpose of the present research is to evaluate the cytotoxicity and the effects on the cell cycle of pure artemisinin and a hydroalcoholic extract obtained from *A. annua* on a canine osteosarcoma cell line (D-17). Considering the suggested crucial role of iron on artemisinin activity, the second aim was addressed to determine the intracellular iron concentration and its possible correlation with the observed effects.

## 2. Materials and Methods

### 2.1. Chemicals and Reagents

All reagents were obtained from Sigma Aldrich (St. Louis, MO, USA), if not otherwise specified, and were ultrapure grade, including 98% pure artemisinin (CAS number: 63968-64-9). Minimum Essential Media (MEM), heat-inactivated fetal bovine serum (FBS), and Dulbecco's phosphate-buffered saline (DPBS) were purchased from Gibco-Life Technologies (Carlsbad CA, USA). A commercial hydroalcoholic extract obtained from *A. annua* and composed of 65% ethanol, 20% of aerial parts, and water was used. This extract contained 2 mg artemisinin/ml (corresponding to 7 mM) as declared by the producer. All plastic supports were purchased from Falcon, Becton-Dickinson (Franklin Lakes, NJ, USA).

### 2.2. Cell Culture and Treatment

The canine osteosarcoma cell line (D-17) was purchased from the “Istituto Zooprofilattico Sperimentale della Lombardia e dell'Emilia Romagna—Sez. Brescia.” Cells were cultured in Minimum Essential Media (MEM) added with 2 mM L-glutamine and FBS (5%) in a 5% CO_2_ atmosphere at 37°C. The first seeding after thawing was always performed in T-75 tissue culture flasks (4 × 10^6^ cells/flask), and subsequent experiments were conducted in T-25 flasks (cell cycle analysis and iron quantification) and in 96-well assay plates (cytotoxicity). Artemisinin was dissolved in DMSO to obtain a 50 mM stock solution then diluted in culture medium to obtain the required concentrations. Hydroalcoholic extract of *A. annua* was directly diluted in culture medium to obtain the required concentrations. For each treatment, the same concentration of the specific vehicle was used as control, DMSO for artemisinin and a solution of 65% ethanol for hydroalcoholic extract. Cells grown in culture medium (MEM + 5% FBS with 2 mM L-glutamine) without treatments were considered “untreated cells.”

### 2.3. Cytotoxicity

1.5 × 10^4^ cells/well were seeded in a 96-well plate and exposed for 24 h to increasing concentration of pure artemisinin (50, 100, 250, 500, 750, 1000, and 2000 *μ*M) or increasing doses of *A. annua* hydroalcoholic extract corresponding to artemisinin concentrations of 14, 35, 70, 140, 280, 420, and 700 *μ*M, on the base of the declared concentration of 2 mg artemisinin/ml in hydroalcoholic extract. For each treatment, the same concentration of the specific vehicle was used as control, DMSO for artemisinin and a solution of 65% ethanol for hydroalcoholic extract. Cytotoxicity was measured using tetrazolium salt (*In Vitro* Toxicology Assay Kit, MTT-based). The formazan absorbance was measured at a wavelength of 570 nm, using Infinite® F50/Robotic Absorbance microplate readers, TECAN (Life Science). The background absorbance of multiwall plates at 690 nm was also measured and subtracted from the 570 nm measurements. The concentrations of artemisinin required for 50% inhibition of cell viability (IC_50_) were calculated by Prism GraphPad software, and the IC_50_ values were used for subsequent experiments.

### 2.4. Cell Cycle Analysis

For the analysis of cell cycle in flow cytometry, after 24 h of treatment, aliquots of 1 × 10^6^ cells in duplicate for each treatment and for the specific vehicle used as control (IC_50_ standard artemisinin, IC_50_*A. annua* hydroalcoholic extract, the same concentration of DMSO for artemisinin and a solution of 65% ethanol for hydroalcoholic extract) were washed from growth medium by centrifuging at 240 x g for ten minutes. Then, the resulting pellet was resuspended in 1 ml of a solution containing 0.1% sodium citrate, 0.1% Nonidet, 10 *μ*g/ml of RNAse, and 50 *μ*g/mL of propidium iodide (final concentration 1 × 10^6^ cells/ml). After 30 min at 37°C in the dark, the isolated nuclei were analysed by using a Bryte HS flow cytometer (Bio-Rad) equipped with a Xe/Hg lamp and a filter set to obtain an excitation at 488 nm. PI fluorescence was collected on a linear scale at 600 nm, and the DNA distribution was analysed by the ModFit software (Verity, USA). For microscopical evaluation, cells (7000 cells/well) were seeded on an eight-well chamber slide (BD Falcon, Franklin Lakes, NJ). After 24 h of treatment, cells were stained with Hoechst 33342 15 *μ*g/ml for 30 minutes in a 5% CO_2_ incubator at 37°C. After 3 washes in DPBS, cells were analysed using an Eclipse E600 epifluorescence microscope equipped with a Nikon digital camera and the ACT-2U software for image capturing (Nikon, Tokyo, Japan). Images were analysed by counting a minimum of 400 nuclei in order to evaluate the percentage of apoptotic ones.

### 2.5. Iron Quantification

For the iron determination, a Spectra AA-20 atomic absorption spectrometer (Varian) equipped with a GTA-96 graphite tube atomizer and the sample dispenser was used. The optimization of the analytical method was obtained following Tüzen [12] with minor changes. The graphite tubes employed were coated GTA tubes (Agilent Technologies, Germany), the hollow cathode lamp current was 7 mA, and measurements were performed at 248.3 nm resonance lines using a spectral slit width of 0.2 nm. During spectrophotometer readings, the internal argon flow rate in the partition graphite tubes was maintained at 300 ml/min and was interrupted in the atomization phase. Ramp and hold times for drying, pyrolysis, atomization, and cleaning temperatures were optimized to obtain maximum absorbance without significant background absorption; therefore, background correction was not necessary.

The calibration curve was obtained by diluting 1 mg/ml standard stock solution of iron (BDH Chemicals, Poole, England) with Milli-Q water to obtain working standards containing 0, 20, 40, and 60 ng/ml of iron and by plotting the absorbance at 248.3 nm against iron concentrations. The equation of the curve was *y* = 0.0109*x*, and the calculated regression coefficient (*r*) was 0.993. The method was validated with standard reference material (BDH Chemicals, Poole, England), and the accuracy of the method, calculated as the percentage of the certified value, resulted to 105%.

For the quantification of intracellular iron, cells were grown in a T-75 flask (4 × 10^6^ cells) until confluence. Then, cells were treated with IC_50_ standard artemisinin and IC_50_*A. annua* hydroalcoholic extract, for 24 h. For each treatment, the same concentration of the specific vehicle was used as control, DMSO for artemisinin and a solution of 65% ethanol for hydroalcoholic extract. After that, aliquots of 1 × 10^6^ cells for each treatment were harvested, counted, and centrifuged at 800 x g for 10 min. The pellet was washed twice with DPBS and then resuspended in a solution of 1 M HNO_3_ at a final concentration of 1 × 10^6^ cells/ml, digested at room temperature until completely dissolved, and finally used for iron quantification as reported by Sargenti et al. [13]. The detection limit (LOD), defined as the concentration corresponding to 3 times the standard deviation of 6 blanks, was 0.8 ng/ml. Iron concentration is reported as ng Fe/1 × 10^6^ cells.

### 2.6. Statistical Analysis

Data for MTT and iron were analysed with a one-way analysis of variance (ANOVA) followed by *post hoc* Dunnett's multiple comparison test. Data of the cell cycle were analysed with two-way analysis of variance (ANOVA) followed by the Bonferroni multiple comparisons. Differences of at least *p* < 0.05 were considered significant. Statistical analysis was carried out using Prism GraphPad software.

## 3. Results

### 3.1. Effect of *Artemisia annua* Hydroalcoholic Extract and Artemisinin on Cell Viability

**Figure 1:**
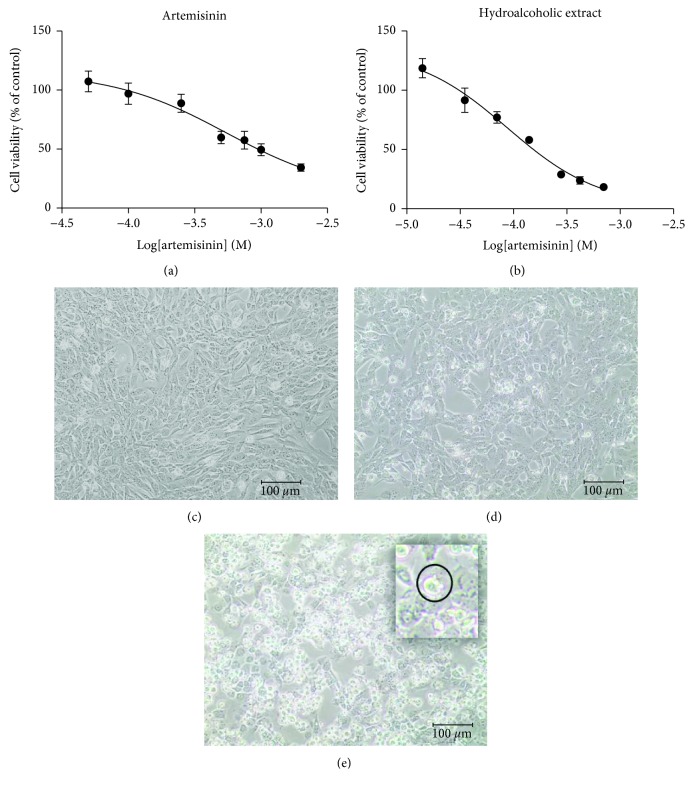
Effect of artemisinin and *A. annua* hydroalcoholic extract on D-17 cells. Dose-response curve: D-17 cell viability upon treatment with different concentrations of (a) pure artemisinin and (b) *A. annua* hydroalcoholic extract. For each treatment, the same concentration of the specific vehicle was used as the control, DMSO for artemisinin and a solution of 65% ethanol for hydroalcoholic extract. Representative images of D-17 cell morphology: (c) untreated cells and in the presence of (d) artemisinin and (e) hydroalcoholic extract, to note the extensive presence of cells with rounded morphology and condensation of cytoplasmic constituents. Dose-response curves are reported as the mean ± SD from two independent experiments (*n* = 2), each performed in sextuple.

The effect of artemisinin and *A. annua* hydroalcoholic extract was evaluated on D-17 cells by the MTT assay. Both artemisinin and hydroalcoholic extract induced a decrease of cell viability at all concentrations and exerted cytotoxic effect in a dose-dependent manner (Figures 1(a) and 1(b)). After 24 h, both products provoked an increased number of detached cells with round shape and condensation of cytoplasmic constituents, more evident in the presence of hydroalcoholic extract (Figures 1(d) and 1(e)). Data obtained from MTT analyses were elaborated to assess the concentration of artemisinin required for 50% inhibition of cell viability (IC_50_): the values corresponded to 548 *μ*M for the standard and 65 *μ*M for the hydroalcoholic extract (Figures 1(a) and 1(b)).

### 3.2. Effect of *Artemisia annua* Hydroalcoholic Extract and Artemisinin on the Cell Cycle

**Figure 2:**
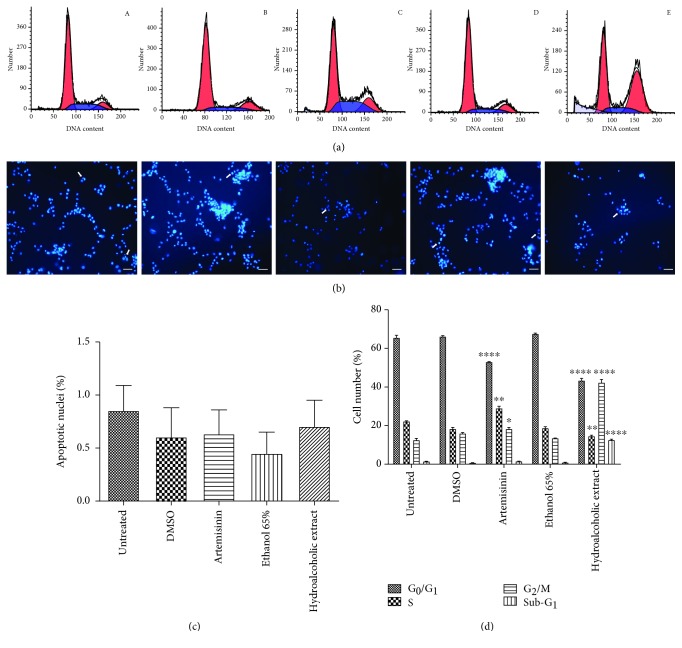
Representative image of cell cycle analysis (a) and nuclear staining with Hoechst 33342 (b) of D-17 cells after 24 hours of treatment: (A) untreated cells and in the presence of (B) DMSO, (C) 548 *μ*M artemisinin, (D) a solution of 65% ethanol, and (E) hydroalcoholic extract corresponding to 65 *μ*M artemisinin. (c) Percentage of apoptotic nuclei. (d) Cell cycle distribution. Data are reported as the mean ± SD (*n* = 2). Significant differences *vs.* untreated cells, comparing cell cycle phases (G_0_/G_1_, S, G_2_/M, and Sub-G_1_), are indicated by ^∗^*p* < 0.05 and by ^∗∗^*p* < 0.01 and ^∗∗∗∗^*p* < 0.0001 (two-way ANOVA followed by the Bonferroni multiple comparisons). Arrows indicate apoptotic nuclei. Scale bar = 50 *μ*m.

The effect of *A. annua* hydroalcoholic extract and artemisinin on the D-17 cell cycle was evaluated by flow cytometry, and data were analysed with ModFit software. Untreated cells presented a typical cytogram of a diploid cell population (Figures 2(a) A and 2(d)). As shown in Figures 2(a) C and E and 2(d), significant changes in the D-17 cell cycle were determined in the presence of both pure artemisinin and hydroalcoholic extract. A significant decrease (*p* < 0.0001) of cells in the G_0_/G_1_ phase was observed after both treatments. Pure artemisinin caused a significant increase (*p* < 0.01) of cells in the S phase, while hydroalcoholic extract induced a significant (*p* < 0.0001) increase of cells in the G_2_/M phase. The pattern of the DNA distribution revealed only in the samples treated with the hydroalcoholic extract a significant increase of a sub-G1 population. DNA fragments of very variable measures indicated the presence of debris, typical of a necrotic death rather than a tight sub-G1 peak suggestive of apoptosis. These results were also supported by the microscopic examination of the cells stained with the vital nuclear stain Hoechst 33342, where almost no fragmented nuclei, a distinct morphological mark of apoptosis, were detected (Figures 2(a) E and 2(c)). The vehicles, DMSO and ethanol 65%, did not significantly affect the cell cycle (Figures 2(a) B and D and 2(d)).

### 3.3. Effect of *Artemisia annua* Hydroalcoholic Extract and Artemisinin on Intracellular Iron

**Figure 3:**
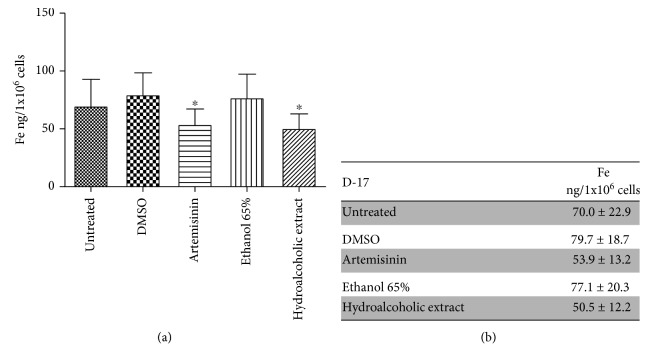
Iron intracellular concentration in D-17 cells measured by atomic absorption spectrometer. For each treatment, the same concentration of the specific vehicle was used as the control, DMSO for artemisinin and a solution of 65% ethanol for hydroalcoholic extract. Data are reported as the mean ± SD from three independent experiments (*n* = 3), each performed in quintuplicate. Significant differences *vs.* untreated cells are indicated by ∗ (*p* < 0.05 one-way ANOVA followed by post hoc Dunnett's multiple comparison test).

Iron concentrations in D-17 cells were reported in Figure 3. Intracellular mean iron concentration in untreated D-17 cells was 70 ± 22.9 ng/1 × 10^6^ cells. Cells exposed to pure artemisinin and *A. annua* hydroalcoholic extract had significantly lower concentrations of intracellular iron than the untreated cells (*p* < 0.05) (Figures 3(a) and 3(b)). The cells treated with the extract had a lower concentration of iron than those treated with pure artemisinin; this difference was not statistically significant. The intracellular iron concentration of cells exposed to the same concentration of the specific vehicle, DMSO for artemisinin and a solution of 65% ethanol for hydroalcoholic extract, was not statistically different from that of the untreated cells.

## 4. Discussion

Osteosarcoma (OSA) is the most common primary bone tumor in humans and in dogs and is characterized by locally aggressive and highly metastatic behaviour [8, 9]. The development of new drugs is necessary to improve the therapeutic outcome, in particular in the presence of multiple resistance and metastatic OSA. Therefore, the first aim of this study was to investigate the cytotoxic effect of artemisinin in comparison with a hydroalcoholic extract obtained from *A. annua*, on a canine osteosarcoma cell line (D-17). Our results demonstrated the cytotoxic effect of both artemisinin and hydroalcoholic extract in a dose-dependent manner. In particular, IC_50_ for artemisinin corresponded to 548 *μ*M whilst for the hydroalcoholic extract the value was significantly lower (65 *μ*M). For artemisinin, the obtained values are in the range of those reported in human tumor cell lines; in fact, Efferth et al. [14] reported a wide range of IC_50_ values of pure artemisinin for a panel of different human cell lines, from 57.1 *μ*M for leukaemia cells to 1602 *μ*M for HeLa cells. In 2014, Jirangkul et al. [15] reported IC_50_ values of pure artemisinin for two human osteosarcoma cell lines, MG63 and 148B, with IC_50_ of 167 *μ*M and 178 *μ*M, respectively. To our knowledge, only dihydroartemisinin (DHA) cytotoxicity was evaluated on canine OSA cell lines. In particular, Hosoya et al. [16] investigated the cytotoxic effect of DHA on four canine OSA cell lines, D-17, OSCA2, OSCA16, and OSCA50, reporting IC_50_ values of 8.7, 43.6, 16.8, and 14.8 *μ*M, respectively.

In accordance with Efferth et al. [14], who reported in HeLa cells that *A. annua* extract is more cytotoxic than pure artemisinin, our results indicated an IC_50_ for the plant extract one order of magnitude lower than pure artemisinin. The same authors tested fourteen extracts of seven different *A. annua* preparations with different phytogeographical origins. They extracted plants in dichloromethane or methanol and evaluated the activity of the extracts on HeLa cells, obtaining IC_50_ values ranging from 54.1 to 275.5 *μ*g/ml for dichloromethane extracts and from 276.3 to 1540.8 *μ*g/ml for methanol extracts. A phytochemical investigation of the extracts by GLC-MS revealed the presence of artemisinin, arteanuine B, and scopoletin in all extracts, confirming *in vitro* the synergistic effect of the mixture of compounds that constitute the phytocomplex. Breuer and Efferth [17] in 2014 reported the successful use of *Herba A. annua Luparte®* as adjuvant therapy for veterinary sarcoma treatment. They found that this extract contained a high amount of scopoletin, while artemisinin represented just a minor component. Scopoletin could contribute to the anticancer activity of the phytoextract analysed in this study as suggested by other authors [18]. It has also been reported that an ethanolic extract from *Artemisia nilagirica* which does not contain artemisinin showed anticancer activity [19]. Therefore, it seems unlikely that the significantly higher cytotoxic effect of the hydroalcoholic extract is due to artemisinin only but more probably it is due to a synergistic effect of different molecules of the phytocomplex, which deserves more attention and careful characterization in future studies.

In the literature, heterogeneous results are reported on the action of artemisinin and its derivatives on the cell cycle arrest. It has been reported that artemisinin and its derivatives cause cell cycle arrest mainly in the G_0_/G_1_ phase through downregulation of cyclin E, cyclin D1, and cyclin-dependent kinases 2 and 4 in several tumor cell lines, including human breast cancer cells [20], gallbladder cancer cell lines [21], neuroblastoma [22], lung carcinoma cells (A549), and nonsmall lung cancer cells (H1299) [23]. In the D-17 canine osteosarcoma cell line, pure artemisinin slightly but significantly increased the number of cells in the S phase, and this has been observed also in EN2 tumor cells by Beekman et al. [24]. On the other hand, the hydroalcoholic extract caused a significant increase of D-17 cells at the G_2_/M phase. Other authors found that artemisinin-derived drugs induced G_2_/M cell cycle arrest. In particular, dihydroartemisinin induced G_2_ arrest in the human osteosarcoma cell line [25], ovarian carcinoma cell line [26], and hepatocellular carcinoma cell line [27], while artesunate induced G_2_ arrest in breast carcinoma cell lines [28], rat pituitary adenoma cell line [29], and kidney carcinoma cell lines [30]. However, the effects of a pure compound could be different from those induced from phytoextracts, whose complexity has to be considered [31]. *A. annua* extracts could contain several molecules, and their composition could be changed not only by the strategy of extraction but also by the time and the place where the plant is harvested [32, 33]. Kim et al. [34] evaluated the effect of an *A. annua* extract on human colon cancer cell line HCT116 and found that cell cycle arrest occurred at the G_1_/S phase mediated by the Akt/mTOR pathway. Later on, they demonstrated that *A. annua* extract induced apoptosis through the regulation of specific proteins such as Bax, Bak, and cytochrome *c* in PDK1/Akt signaling pathways via a PTEN/p53-independent manner [35]. In this work, cell cycle analysis suggested that a modified cellular distribution in different phases of the cycle occurred in the treated cells and that the sub-G1 peak observed was due to cellular debris rather than fragmented DNA, which is typical of apoptosis. The differences in cell cycle phase arrest might be ascribed to the phytochemical complexity of the extract, in which the synergy of the different compounds could lead to alternative molecular interactions hampering one pathway or another. More research is needed to unravel the complex mechanism underlying the effect of *A. annua* extract on the cell cycle.

Often tumor cells present an altered iron metabolism and higher iron intake than normal cells to deal with their enhanced metabolic demand, thus presenting an increased number of transferrin receptors [6, 36, 37]. Therefore, the determination of intracellular iron concentration is a prerequisite for any further analyses and can provide an important support to the involvement of this metal. In the present study, iron concentration determined in untreated D-17 cells falls within the range of those reported by other authors in different mammalian cells [38], though different preparation protocols, analytical methods, and measurement systems hampered in many cases a direct comparison of data. Following the incubation in the presence of pure artemisinin and *A. annua* extract, an alteration of iron metabolism in D-17 cells was determined, namely, a significant decrease of intracellular total iron. On the basis of previous papers, it is well known that, in the presence of ferrous ions, the leading event in the artemisinin-induced cytotoxic cascade is represented by the activation of the molecule by the cleavage of the endoperoxide bridge producing a carbon-centered radical able to induce intracellular oxidative damage [2, 7]. However, the link between the complex molecular machinery which regulates iron metabolism and the anticancer activity of artemisinin and *A. annua* extracts is far from being clarified. When analysing the effect of artemisinin on HeLa cancer cell proteome, Zhou et al. [39] identified as artemisinin targets 79 proteins involved mainly in membrane transport, protein trafficking, cell death and survival, and nucleic acid metabolism. The authors hypothesized that among these protein transferrin receptors, which are overexpressed in several tumor cells [36, 37], could be alkylated and subsequently inactivated by artemisinin, leading to a selective depletion of iron in cancer cells. The decrease of intracellular iron reported in the present research supports this hypothesis and shows additional evidence regarding the alteration of iron metabolism induced by artemisinin and *A. annua* extracts.

The ability of iron to gain and lose electrons makes this transition metal essential for life but also enables iron to participate in potentially deleterious reactions; therefore, the intracellular iron concentrations are finely regulated [40]. In particular, a controlled labile iron pool, e.g., a pool of loosely bound redox-active iron, is present within the cells and serves as a crossroad of intracellular iron metabolism [41]. In normal cells, this pool is maintained within a narrow range of concentrations, while in cancer cells, a reduction of ferritin iron storage can increase this labile iron pool and the risk of oxidative stress, which can ultimately determine the death of the cells [36]. The examination of D-17 cells did not show fragmented nuclei but revealed many dead cells with a clear rounded shape and a “ballooning” phenotype, in particular following the treatment with *A. annua* extract. This phenotype was recently reported as a hallmark of a novel mode of iron-dependent cell death, namely, ferroptosis [42]. Ferroptosis is a regulated cell death characterized by an increase of free intracellular iron followed by an iron-dependent accumulation of lipid hydroperoxides and a specific phenotype morphologically distinct from those associated with apoptosis and necrosis [43]. To date, different types of tumors have shown sensitivity to ferroptosis [44, 45]; however, the role of iron in this new type of cell death is far from being fully elucidated. In treated D-17 cells, it can be hypothesized that the low total intracellular iron, due to the alkylation of transferrin receptors, triggers the activation of ferritin degradation which can occur by either autophagic [46] or lysosomal degradation of the protein [47]. In turn, this event might contribute to increase the labile redox-active iron pool, determining a subsequent iron-driven lipid peroxidation and the activation of ferroptosis. Further analyses are needed to verify this challenging hypothesis.

## 5. Conclusions

Overall, the results reported in this research showed a more potent cytotoxic activity of the hydroalcoholic extract than pure artemisinin on the D-17 canine osteosarcoma cell line, indicating a possible synergistic effect of other bioactive molecules. These findings offer additional evidence on the biological activity of artemisinin and *A. annua* extract for their possible and safer therapeutic use. Moreover, they support the dog as a spontaneous animal model for the study of osteosarcoma. Despite the detection of intracellular total iron by a sensitive and specific technique that showed clear evidence of an alteration of iron metabolism, this paper is affected by limitations, namely, the lack of investigation about the expression of iron-related genes and ROS involvement in ferroptosis. Future researches should therefore be focused on these pivotal topics and on the role of the iron labile pool.

## Data Availability

The data used to support the findings of this study are available from the corresponding author upon request.
